# Exploring the impact of intravenous thrombolysis on length of stay for acute ischemic stroke: a retrospective cohort study

**DOI:** 10.1186/s12913-015-1080-0

**Published:** 2015-09-23

**Authors:** Ling-Chien Hung, Ya-Han Hu, Sheng-Feng Sung

**Affiliations:** Division of Neurology, Department of Internal Medicine, Ditmanson Medical Foundation Chia-Yi Christian Hospital, 539 Zhongxiao Rd, Chiayi City, 60002 Taiwan; Department of Information Management and Institute of Healthcare Information Management, National Chung Cheng University, Chiayi County, Taiwan; Department of Nursing, Min-Hwei Junior College of Health Care Management, Tainan, Taiwan

**Keywords:** Acute ischemic stroke, Classification and regression tree, Intravenous thrombolysis, Length of stay

## Abstract

**Background:**

Understanding the factors that influence the hospital length of stay (LOS) for patients with stroke will help in discharge planning and stroke unit management. We explored how intravenous thrombolysis (IVT) affects LOS in an acute-care hospital setting.

**Methods:**

We analyzed adult patients with ischemic stroke who presented within 48 h of onset from a hospital-based stroke registry. The relationship between IVT and prolonged LOS (LOS ≥ 7 days) was studied by both multivariate logistic regression and the classification and regression tree (CART) analyses.

**Results:**

Among the study population of 3054 patients, 1110 presented within 4.5 h. The median LOS (interquartile range) was 7 (4 to 11) days, and 1619 patients had prolonged LOS. Multivariate logistic regression revealed that IVT (odds ratio, 0.53; 95 % confidence interval 0.38–0.74) was an independent factor that reduced the risk of prolonged LOS, whereas age, National Institutes of Health Stroke Scale (NIHSS) score, diabetes mellitus, and leukocytosis at admission predicted prolonged LOS. CART analysis identified 4 variables (NIHSS score, IVT, leukocytosis at admission, and age) as important factors to partition the patients into six subgroups. The patient subgroup that had an NIHSS score of 5 to 7 and received IVT had the lowest probability (19 %) of prolonged LOS.

**Conclusions:**

IVT reduced the risk of prolonged LOS in patients with acute ischemic stroke. Measures to increase the rate of IVT are encouraged.

## Background

Stroke has been one of the three leading causes of mortality since the1960s in Taiwan [1]. In addition, it is an important cause of disability in adults, particularly among the elderly population, and it imposes a huge burden on health care resources [2, 3]. Its global disease burden increased from fourth in 2001 to third in 2010 [4, 5]. In Taiwan, the stroke-associated disease burden increased each year from 2000 to 2005 and is expected to further increase in the future [6]. Therefore, the health care costs for stroke will inevitably rise. With the continuing growth of health care costs, a hospital global budgeting system has been adopted since July 2002 by the Taiwan National Health Insurance (NHI) to control medical expenditures. Consequently, the overall hospital net revenues have reduced and hospitals are under increased financial stress [7].

The hospital length of stay (LOS) for patients with stroke has been found to be closely correlated with medical costs [2, 8–10]. A better understanding of the factors that influence LOS would not only facilitate discharge planning and help control medical costs but also have implications for the management of stroke units. LOS is a widely used quality measure that is incorporated in Taiwan’s nationwide health care quality indicator system [11]. Several factors have been identified as significant predictors of prolonged LOS for patients with stroke, including initial stroke severity, stroke subtype, leukocytosis at admission, prestroke disability, diabetes mellitus, congestive heart failure, atrial fibrillation, coronary heart disease, chronic obstructive pulmonary disease, and hyperlipemia [9, 12–16]. Validated tools are also available to predict prolonged LOS in the acute stroke setting [14, 17–19].

Intravenous thrombolysis (IVT) with tissue-type plasminogen activator (tPA) is currently the standard treatment for acute ischemic stroke. It significantly reduces the proportion of patients who were dead or dependent at 3 to 6 months after the stroke [20]. However, few reports have investigated the relationship of IVT and LOS. One study showed that among patients treated with IVT, the independent variables that affected LOS include a lack of improvement at 24 h after treatment, cortical involvement, and the presence of new infarction on the 24-h computed tomography scans [13]. Data from randomized trials showed significantly shorter LOS in tPA-treated patients than in control patients [21, 22]. However, whether IVT impacts LOS in patients with acute ischemic stroke in daily practice remained unclear. Therefore, we conducted this study to examine how IVT affects LOS in an acute-care hospital setting using both conventional statistical methods and data mining techniques.

## Methods

### Patients

We analyzed data from a hospital-based stroke registry, which prospectively registered all patients with stroke who were admitted to the Ditmanson Medical Foundation Chia-Yi Christian Hospital according to the design of the nationwide Taiwan Stroke Registry [23]. The hospital, which has 1000 beds and is the largest hospital in Chiayi City, provides health care to approximately one million inhabitants in the city and its adjoining rural area. The Chia-Yi Christian Hospital Institutional Review Board approved the study protocol.

We identified patients with ischemic stroke who were aged 20 years or older and were admitted within 48 h of stroke onset between October 2007 and December 2013. Patients with in-hospital stroke and those without complete data were excluded. Ischemic stroke is defined as the acute onset of neurologic deficits that persist longer than 24 h with no indications of hemorrhage on the first brain computed tomography or with acute corresponding ischemic lesion(s) on diffusion-weighted magnetic resonance imaging. Data on patient characteristics, including demographic data, medical history, comorbidity conditions, treatments, complications, and outcomes, were obtained from the stroke registry. Stroke severity was assessed with the National Institutes of Health Stroke Scale (NIHSS) and stroke mechanism was determined based on the Trial of ORG 10172 in Acute Stroke Treatment classification [24]. The functional status was evaluated at discharge according to the modified Rankin Scale.

The decision to treat stroke patients with IVT was generally made according to the guidelines released by the Taiwan Stroke Society in 2003 [25], which have been adopted by the Taiwan NHI as the basis of reimbursement for IVT. The major discrepancies between the guidelines and the American Heart Association guidelines [26] are the exclusion of patients with the following characteristics: older than 80 years, history of previous stroke and diabetes mellitus, stroke onset more than 3 h, severe stroke (NIHSS score > 25), and minor stroke (specifically defined as a stroke with an NIHSS score < 6). Patients who were not eligible for IVT according to the Taiwan Stroke Society guidelines but who were indicated for treatment according to the American Heart Association guidelines (e.g., age over 80 years, stroke onset between 3 and 4.5 h, minor stroke with an NIHSS score < 6, or severe stroke with an NIHSS score > 25) were treated if the physician and the patient agreed and if the patient paid the cost of tPA on their own.

### Definition of the variables

The outcome of interest was prolonged LOS that was dichotomized at ≥ 7 days of hospitalization, and this division conformed to those of previous studies on LOS in patients with stroke [13, 14]. The study hospital only offers acute-care hospitalization of patients with stroke. LOS was defined as the time from admission to discharge. The admission date was defined as the date on which the patient was admitted to the hospital, and the discharge date was defined as the date on which the patient died or was discharged to home, another hospital, a rehabilitation facility, or a nursing home.

The predictor variables were determined a priori on the basis of previous studies [9, 12–16] and clinical experience. In addition to the use of IVT, we only evaluated those factors that could be assessed at the time of admission. The variables included age, gender, admission NIHSS score, prior functional dependency (modified Rankin Scale score ≥ 3), medical conditions (hypertension, diabetes mellitus, hyperlipidemia, prior stroke, atrial fibrillation, coronary heart disease, congestive heart failure, malignancy, or uremia on dialysis), smoking status, prior use of antiplatelet or oral anticoagulant drugs, and leukocytosis (>10 × 10^9^ cells/L) at admission.

### Statistical analysis

The bivariate associations between the baseline characteristics of the patients and prolonged LOS were examined with appropriate tests (chi-squared test, *t*-test, or Mann–Whitney *U*-test). A multivariate logistic regression was used to determine the variables independently associated with prolonged LOS. All the prespecified predictor variables were entered into the model. Considering the nonlinear relationship between the NIHSS score and LOS [12–14], stroke severity was categorized into the following 5 groups according to the NIHSS score: 0–5, 6–10, 11–15, 16–20, and > 20 [14]. Because of the increased risk of early mortality in patients with the most severe strokes, a separate multivariate logistic regression was conducted only on hospital survivors. The discrimination of the model was assessed with the area under the receiver operating characteristic curve (AUC), and the model fit was evaluated with the Hosmer–Lemeshow goodness-of-fit statistic. Because IVT should be administered within a narrow time window, we conducted a subgroup analysis of patients who presented to the hospital within 4.5 h of stroke onset. Although LOS was dichotomized in the present study, we conducted additional analyses treating LOS as a continuous variable. Since LOS shows a right skewed distribution, the natural logarithm of LOS was used as the dependent variable for the multiple linear regression analyses. General statistical analyses were performed with Stata 13 (StataCorp LP, College Station, TX, USA). Two-tailed *p* values less than 0.05 were considered statistically significant.

To stratify the risk of prolonged LOS, all the variables of interest were included in classification and regression tree (CART) analysis for generating a binary decision tree. The CART algorithm grows a tree from the root by selecting the best predictor variable, which is the one with the lowest Gini index value, as an internal node. The patients are then split into two subgroups on the basis of the value of the selected variable [27, 28]. The partitioning process is recursively applied until the stopping criteria are fulfilled or splitting is impossible. A class label is then assigned to the terminal node (i.e., leaf node) on the basis of majority voting. After a decision tree is fully grown, CART analysis conducts a minimal cost-complexity pruning method to avoid overfitting. We used the simpleCART module in Weka 3.6.11 open-source data mining software (www.cs.waikato.ac.nz/ml/weka) to generate a CART tree. The minimum number of cases in each node was set to 30 to simplify the tree structure. A 10-fold cross validation was used to estimate the predictive accuracy and AUC of the tree model.

## Results

After excluding the 105 patients who had in-hospital stroke and the 92 patients with missing data, 3054 patients were used in the analysis. The in-hospital mortality was 3 %. The median LOS (interquartile range) was 7 (4 to 11) days, and the LOS was ≥ 7 days in 1619 (53 %) patients. Table 1 summarizes the demographic and clinical characteristics of the patients. Compared with the patients who were hospitalized for less than 7 days, those with prolonged LOS were older and more likely to be female, and they had more severe strokes, less strokes due to small-vessel occlusion, and a higher prevalence of hypertension, diabetes mellitus, prior stroke, atrial fibrillation, coronary heart disease, and congestive heart failure. In addition, they were prone to be functionally dependent before admission, have leukocytosis at admission, be treated with IVT, and have more complications during the hospitalization. Although the patients with prolonged LOS had worse functional outcomes at discharge, their in-hospital mortality rate did not differ from that of the patients without prolonged LOS.

**Table 1:** Characteristics of patients by LOS category

Characteristic	LOS < 7 days (*n* = 1435)	LOS ≥ 7 days (*n* = 1619)	*P* value
Demographics			
Age, mean (SD), y	67.8 (11.9)	70.8 (12.0)	< 0.001
Female	538 (37.5)	705 (43.6)	0.001
NIHSS at admission			< 0.001
≤ 5	1003 (69.9)	553 (34.2)	
6–10	290 (20.2)	421 (26.0)	
11–15	49 (3.4)	205 (12.7)	
16–20	32 (2.2)	163 (10.1)	
> 20	61 (4.3)	277 (17.1)	
Median (IQR)	4 (2-6)	8 (4-16)	< 0.001
Stroke mechanism			< 0.001
Large artery atherosclerosis	252 (17.6)	515 (31.8)	
Cardioembolism	127 (8.9)	303 (18.7)	
Small-vessel occlusion	717 (50.0)	397 (24.5)	
Specific etiology	11 (0.8)	30 (1.9)	
Undetermined etiology	328 (22.9)	374 (23.1)	
Medical conditions			
Hypertension	1133 (79.0)	1328 (82.0)	0.032
Diabetes mellitus	584 (40.7)	750 (46.3)	0.002
Hyperlipidemia	837 (58.3)	883 (54.5)	0.035
Prior stroke	380 (26.5)	547 (33.8)	< 0.001
Atrial fibrillation	188 (13.1)	372 (23.0)	< 0.001
Coronary heart disease	176 (12.3)	241 (14.9)	0.035
Congestive heart failure	56 (3.9)	118 (7.3)	< 0.001
Malignancy	75 (5.2)	111 (6.9)	0.060
Uremia on dialysis	22 (1.5)	36 (2.2)	0.163
Current smoker	369 (25.7)	351 (21.7)	0.009
Dependent (mRS ≥ 3) before admission	107 (7.5)	266 (16.4)	< 0.001
Prior medication use			
Antiplatelet	352 (24.5)	446 (27.6)	0.058
Oral anticoagulant	38 (2.7)	49 (3.0)	0.530
Leukocytosis (> 10 × 10^9^ cells/L) at admission	215 (15.0)	398 (24.6)	< 0.001
Intravenous thrombolysis	83 (5.8)	123 (7.6)	0.046
Complications			
Pneumonia	16 (1.1)	239 (14.8)	< 0.001
Urinary tract infection	35 (2.4)	212 (13.1)	< 0.001
Upper gastrointestinal bleeding	22 (1.5)	171 (10.6)	< 0.001
Symptomatic intracerebral hemorrhage	4 (0.3)	37 (2.3)	< 0.001
mRS ≥ 3 at discharge	488 (34.0)	1279 (79)	< 0.001
Mortality at discharge	46 (3.2)	45 (2.8)	0.489

Data are numbers (percentage) unless specified otherwise

*IQR* interquartile range, *LOS* length of stay, *mRS* modified Rankin Scale, *NIHSS* National Institutes of Health Stroke Scale, *SD* standard deviation

Table 2 summarizes the results of multivariate logistic regression analysis. Age, admission NIHSS score, diabetes mellitus, and leukocytosis at admission predicted prolonged LOS, whereas the use of IVT was an independent predictor of shorter LOS. Compared with patients with NIHSS scores ≤ 5, the odds ratio of having prolonged LOS increased from 2.75 for patients with NIHSS scores of 6–10 to 8.01 for patients with NIHSS scores of 11–15 and 9.65 for patients with NIHSS scores of 16–20 and then decreased to 7.62 for patients with NIHSS scores > 20. When only hospital survivors were analyzed, the odds ratio increased with increasing NIHSS scores (Table 2). The model discrimination was acceptable with AUC of 0.735 (95 % confidence interval [CI], 0.717–0.752) for all the patients and 0.750 (95 % CI, 0.732–0.767) for the hospital survivors. The Hosmer–Lemeshow goodness-of-fit statistic was not significant for all the patients (*p* = 0.782) and the hospital survivors (*p* = 0.978), indicating adequate fitness. In the subgroup of patients who presented within 4.5 h of stroke onset, the use of IVT remained an independent predictor of shorter LOS (Table 3). In addition, patients taking oral anticoagulants had a lower chance of prolonged LOS. AUC was 0.734 (95 % CI, 0.705–0.764) for all the patients in the subgroup and 0.765 (95 % CI, 0.736–0.793) for the hospital survivors, and the *p* values for the Hosmer–Lemeshow goodness-of-fit tests were 0.856 and 0.424, respectively. In the additional multiple linear regression analyses, the use of IVT was still an independent predictor of shorter LOS in all study patients (*p* = 0.012) and in patients presented within 4.5 h (*p* = 0.034).

**Table 2:** Predictors of prolonged LOS using multivariate logistic regression

	OR (95 % CI), all patients, *n* = 3054	*P* value	OR (95 % CI), hospital survivors, *n* = 2963	*P* value
Age	1.01 (1.00–1.02)	0.019	1.01 (1.00–1.02)	0.019
NIHSS at admission				
≤ 5	Reference		Reference	
6–10	2.75 (2.28–3.33)	< 0.001	2.83 (2.34–3.43)	< 0.001
11–15	8.01 (5.67–11.31)	< 0.001	8.32 (5.86–11.83)	< 0.001
16–20	9.65 (6.32–14.74)	< 0.001	11.07(7.06–17.36)	< 0.001
> 20	7.62 (5.48–10.60)	< 0.001	19.15 (12.01–30.53)	< 0.001
Diabetes mellitus	1.30 (1.10–1.53)	0.002	1.34 (1.13–1.58)	0.001
Leukocytosis (> 10 × 10^9^ cells/L) at admission	1.46 (1.19–1.80)	< 0.001	1.55 (1.25–1.92)	< 0.001
Intravenous thrombolysis	0.53 (0.38–0.74)	< 0.001	0.49 (0.34–0.71)	< 0.001

*CI* confidence interval, *LOS* length of stay, *NIHSS* National Institutes of Health Stroke Scale, *OR* odds ratio

**Table 3:** Predictors of prolonged LOS in patients presented within 4.5 h using multivariate logistic regression

	OR (95 % CI), all patients, *n* = 1110	*P* value	OR (95 % CI), hospital survivors, *n* = 1053	*P* value
NIHSS at admission				
≤ 5	Reference		Reference	
6–10	2.52 (1.80–3.54)	< 0.001	2.75 (1.94–3.89)	< 0.001
11–15	8.69 (5.02–15.0)	< 0.001	9.92 (5.59–17.61)	< 0.001
16–20	8.70 (4.80–15.8)	< 0.001	10.12 (5.36–19.10)	< 0.001
> 20	5.82 (3.69–9.18)	< 0.001	16.99 (8.97–32.19)	< 0.001
Atrial fibrillation	1.37 (0.95–1.97)	0.087	1.68 (1.12–2.51)	0.011
Oral anticoagulant	0.39 (0.18–0.83)	0.015	0.30 (0.13–0.69)	0.005
Leukocytosis (> 10 × 10^9^ cells/L) at admission	1.50 (1.07–2.11)	0.020	1.59 (1.10–2.29)	0.013
Intravenous thrombolysis	0.48 (0.33–0.71)	< 0.001	0.42 (0.27–0.64)	< 0.001

*CI* confidence interval, *LOS* length of stay, *NIHSS* National Institutes of Health Stroke Scale, *OR* odds ratio

**Fig. 1:**
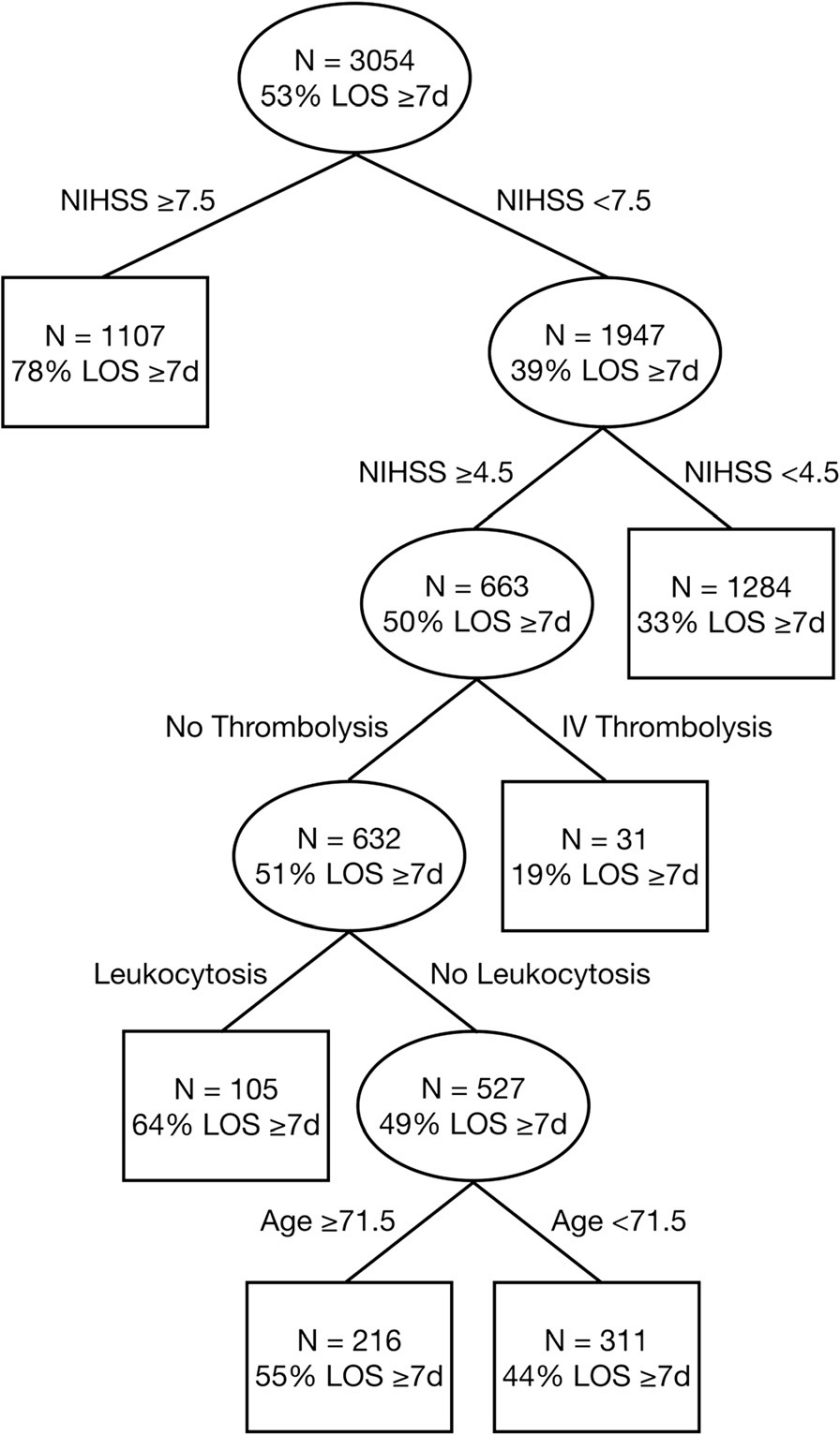
Risk stratification for prolonged LOS by means of the classification and regression tree analysis. Abbreviations: IV, intravenous; LOS, length of stay; NIHSS, National Institutes of Health Stroke Scale

Four variables (admission NIHSS score, use of IVT, leukocytosis at admission, and age) were identified by CART analysis as important factors, which were used to partition the patients into six subgroups (Fig. 1). The NIHSS score was determined as the best discriminator between patients with prolonged LOS and those without it. Patients with an initial NIHSS score ≥ 7.5 had a 78 % probability of prolonged hospitalization. Interestingly, for patients with an NIHSS score < 7.5, the next best predictor was still the NIHSS score. Patients with an NIHSS score < 4.5 had a 33 % risk of prolonged LOS. For patients with an NIHSS score ≥ 4.5 and < 7.5 (i.e., 5, 6, and 7), the use of IVT was the best discriminator in determining prolonged LOS. The subgroup of patients who had NIHSS scores of 5 to 7 at admission and who received IVT had the lowest chance (19 %) of prolonged LOS. When we restricted the analysis to patients with an NIHSS score of 6 or 7 to conform to the reimbursement criteria of the Taiwan NHI, 20 % of patients treated with IVT had prolonged LOS and 54 % of those not treated had prolonged LOS. The evaluation results of the 10-fold cross validation showed that the outcomes of 67.4 % of the patients were correctly predicted by the CART tree, and the estimate of AUC of the tree model was 0.701.

## Discussion

With the use of both logistic regression and CART analyses, we found that IVT independently predicted a lower probability of prolonged LOS during acute-care hospitalization in patients with ischemic stroke who were admitted within 48 h of stroke onset. In addition, the use of IVT was the best determinant of prolonged LOS in patients with an initial NIHSS score of 5 to 7 according to CART analysis. Both analytic methods identified the initial NIHSS score as the major predictor of LOS. Other significant factors were age, diabetes mellitus, and leukocytosis at admission. Although patients with the most severe strokes are at an increased risk of early mortality, the effect of IVT on LOS did not change materially when only hospital survivors were analyzed. In the subgroup of patients who presented within 4.5 h, we identified oral anticoagulant use as an additional factor that reduced the risk of prolonged LOS. While preadmission oral anticoagulant treatment in patients with atrial fibrillation is associated with less severe strokes [29], understanding its relationship with LOS requires further investigation.

In agreement with the results of previous randomized trials [21, 22], our results demonstrated that IVT reduced the chance of prolonged LOS in real-world practice. Moreover, the results showed that patients with NIHSS scores of 5 to 7 had the lowest probability of prolonged LOS if they were treated with IVT, even lower than patients with NIHSS scores < 5. The clinical implications of these findings are twofold if LOS for stroke is to be shortened. First, because IVT is only indicated for patients presenting within a narrow time window, it is crucial for health care providers to promote public awareness of the benefits of prompt stroke treatment. A study has shown that educating patients to seek treatment sooner needs to be a major element of system-wide interventions that are designed to increase the use of thrombolysis treatment in patients with acute ischemic stroke [30]. In Taiwan, the prolonged time interval between the onset of stroke symptoms and the decision to seek medical help is the underlying cause of delayed hospital presentation, particularly in older patients and those with mild stroke [31]. Therefore, public education programs should be targeted to the older population and should emphasize on the recognition of early stroke symptoms. Second, because IVT is not reimbursed by the Taiwan NHI in patients with NIHSS scores < 6, it is prudent for the Taiwan NHI administration to reconsider its coverage policy for IVT.

Our findings were in line with those of earlier studies [12–14] that indicated the important role of the initial NIHSS score in determining LOS and the nonlinear relationship between the NIHSS score and LOS. In our study, increasing NIHSS scores initially increased the chance of prolonged LOS, which then peaked between NIHSS scores of 16 and 20 and then declined, while the risk of prolonged LOS in the hospital survivors was associated with increasing NIHSS scores. In addition, CART analysis automatically determined the optimal cut-off points of NIHSS scores that can be used to guide the need for intervention when assessing a patient’s risk of prolonged LOS. The ability to decide cut-off points is one of the main advantages of decision tree-based methods [32]. In addition, decision tree-based methods facilitate the identification and interpretation of complex interactions among predictor variables [32, 33]. For example, the benefit of IVT, in terms of reducing the risk of prolonged LOS, could hardly be identified in patients with NIHSS scores of 5 to 7 in logistic regression analysis.

It is generally agreed that LOS is the main cost-determining factor in patients with acute stroke [2, 8–10]. However, shortened LOS cannot be necessarily translated to a reduction in acute-care hospital costs for patients treated with IVT because of the extra cost that is incurred by administering tPA. Nevertheless, because IVT significantly reduces the probability of patients being functionally dependent after stroke [20] and level of physical disability and level of neurological deficit determine direct healthcare costs within 1 year [34], the financial benefits of IVT are mainly related to the decreased costs in long-term hospital and community care [35]. A systematic review found that IVT was associated with an acceptable rise in short-term costs in the management of patients with acute ischemic stroke and it was cost saving in the long run [36]. Even without reducing the acute-care hospital costs, the reduced incidence of prolonged LOS as a result of IVT is still meaningful with regard to the allocation of health care resources. With the rapid growth in the elderly population in Taiwan [37], the incidence of stroke will substantially increase and the need for acute stroke care will grow. Shortening acute-care LOS might help relieve the demand for stroke unit beds and neurologists dedicated to stroke care. In particular, a growing shortage of vascular neurologists is anticipated [38].

The limitations of our study are as follows: First, this is a single-hospital study, and generalizations should therefore be made with caution. Second, given the retrospective nature of the study and the use of register data, not all variables that may influence LOS were available for analysis. For instance, we did not explore IVT-related variables such as rapid improvement after thrombolysis, new infarction, and cortical involvement on follow-up neuroimaging, which have been shown to independently affect LOS [13]. Third, we did not investigate in-hospital neurological or medical complications, which are associated with longer LOS [39]. We evaluated only those factors that could be assessed at the time of admission because tPA should be administered shortly after patient arrival at the hospital.

## Conclusions

IVT decreased the risk of prolonged LOS in patients with acute ischemic stroke regardless of inital stroke severity. Shortening acute-care LOS could help reduce the demand for stroke unit beds and thus improve health care resource allocation. Measures to increase the use of IVT, including public education for stroke awareness and modification of reimbursement criteria for IVT in patients with acute ischemic stroke, are encouraged.

## Abbreviations

AUC: Area under the receiver operating characteristic curve
CART: Classification and regression tree
IVT: Intravenous thrombolysis
LOS: Length of stay
NIHSS: National Institutes of Health Stroke Scale
tPA: Tissue-type plasminogen activator
